# Navigating the trials of a trial: lessons from ProRIDE on recruitment, retention, and follow-up in rural Africa

**DOI:** 10.1186/s13063-026-09447-3

**Published:** 2026-01-15

**Authors:** Sabrina John Moyo, Museveni Justine, Bjørn Blomberg, Iren Høyland Löhr, Joshua Gideon, Paschal Mdoe, Estomih Mduma, Joel Manyahi, Veronika Kuchařová Pettersen, John Paschal, Heidi Syre, Rehema Bukhay, Claus Klingenberg, Nina Langeland

**Affiliations:** 1 Department of Clinical Science, University of Bergen, Bergen, Norway; 2 Department of Tropical Disease Biology, Liverpool School of Tropical Medicine, Liverpool, UK; 3 Department of Paediatrics, Haydom Lutheran Hospital, Mbulu, Manyara Tanzania; 4 Haydom Global Health Research Centre, Haydom Lutheran Hospital, Haydom, Tanzania; 5 Research Group for Child and Adolescent Health, Faculty of Health Sciences, UiT The Arctic University of Norway, Tromsø, Norway; 6 National Centre for Tropical Infectious Diseases, Haukeland University Hospital, Bergen, Norway; 7 Department of Medical Microbiology, Stavanger University Hospital, Stavanger, Norway; 8 Department of Microbiology and Immunology, Muhimbili University of Health and Allied Sciences, Dar Es Salaam, Tanzania; 9 Host-Microbe Interaction Research Group, Department of Medical Biology, UiT The Arctic University of Norway, Tromsø, Norway; 10 Department of Paediatrics and Adolescent Medicine, University Hospital of North Norway, Tromsø, Norway; 11 Norwegian Institute of Public Health, Oslo, Norway

## Abstract

The ProRIDE randomized clinical trial (RCT) started participant recruitment in February 2022, successfully enrolling 2,000 infants within an 11-month period and achieving a follow-up rate of 97% at six months of age. This narrative article describes critical components of the research protocol, the composition of the research team, community sensitization efforts, and the local performance of the study. The research initiative was conceptualized by a collaborative group of scientists from both high- and low-income countries, and its successful implementation was contingent upon the active involvement and participation of a rural, low-income community. Based on previous study findings in Tanzania, which consistently indicated a high prevalence of severe infections related to multidrug-resistant bacteria, the research team recognized the urgent need for preventive strategies aimed at reducing the incidence of sepsis and severe bacterial infections. Given the scarcity of data from rural areas and the imperative for inclusivity, the rationale for conducting this RCT in a rural context was compelling. We believe that a key factor in the successful completion of this trial was the intentional design of a simple, straightforward, and practical intervention, and study framework. Caregivers administered the study medication at home, thereby mirroring real-world therapeutic practices and enhancing the generalizability of the findings. To ensure adherence to the one-month intervention regimen, the research team conducted a day-seven home visit to confirm proper administration of the investigational product and address any associated early challenges. During this interaction, the field workers reiterated the instructions for the proper application of the product, thereby serving as a reminder to the caretaker regarding its correct usage. Additionally, a thorough planning phase was undertaken prior to the study's commencement, involving extensive collaboration between the researchers from Norway, Muhimbili University of Health and Allied Sciences, Tanzania, and the leadership at Haydom Lutheran Hospital. Through a series of meetings and discussions, the research team in Norway and Tanzania identified specific areas requiring enhancement, particularly in laboratory infrastructure and the training of personnel in blood culture techniques and antimicrobial susceptibility testing. Despite facing numerous challenges both prior to and during the trial, this RCT successfully recruited 2,000 participants within 11 months. This accomplishment can be attributed to the strong collaboration and teamwork exhibited throughout the process. The insights gained from this study may be of particular interest to researchers and scientists aiming to conduct investigations involving infants and children in low-income settings.

**Trial registrations** This trial is registered with ClinicalTrials.gov, NCT04172012. November 21, 2019.

## Preface

In this article we describe methodological and practical aspects of our randomised clinical trial (RCT) entitled “Home administration of a multistrain probiotic once daily for four weeks to newborn infants in Tanzania (ProRIDE): a double-blind, placebo-controlled, randomised trial”. The trial protocol and the main results have been published [1, 2]. The primary objective of the ProRIDE trial was to examine whether administration of probiotics for the first four weeks of life, compared to placebo, could reduce morbidity and mortality during the first six months of life. We conducted this trial in a rural setting in Northern Tanzania, where potential benefits of probiotics had not been studied. We successfully recruited and randomized (probiotics vs placebo 1:1) 2,000 infants within just 11 months and maintained a high retention rate of 97% at the 6-month follow-up—underscoring the feasibility of conducting large-scale, high-quality randomized clinical trials in low-resource environments. The daily administration of a multistrain probiotic mixture in the first 4 weeks of life did not reduce the rate of death or hospitalisation up to age 6 months among infants in Tanzania. The proportion of participants with gut carriage of extended-spectrum beta-lactamase-producing Enterobacterales (ESBL-E) at age of 6 weeks was significantly lower in the probiotic group (18%) than in the placebo group (23%) [2]. Despite the logistical and infrastructural challenges in such settings, the probiotic intervention did not lead to short-term safety concerns.

The purpose of this article is to illuminate the often-overlooked background efforts that contribute to the successful execution of a clinical trial in a low-income country. While it may seem like an overstatement to say that “without Haydom, there would be no ProRIDE,” the truth is not far off. An idea conceived by scientists from both high- and low-income countries could only be fully realized through the close involvement and active participation of a rural, low-income community. It is in such settings—where infrastructure is limited, but where trust, collaboration, and commitment run deep—that scientific ideas are brought to life and transformed into meaningful research. The lessons learned and capacities built through this trial in rural Tanzania not only helped answer a pressing global health question but also demonstrated how high-quality research in resource-limited settings can yield insights relevant far beyond their borders.

### How it started and reason for the research question

The collaboration between the Norwegian and Tanzanian partners has a 25-year long history, involving educational and scientific collaboration. The collaboration is based on the systematic institutional collaboration between the Centre for International Health at the University of Bergen (UiB), Norway and Muhimbili University of Health and Allied Sciences (MUHAS) in Dar es Salaam, the biggest city in Tanzania. This collaboration has yielded significant outcomes, including specialized clinical training programs and doctoral degrees, evidenced by numerous scientific publications [3–9]. A partnership over 15 years between Stavanger University Hospital (SUH), Norway and Haydom Lutheran Hospital (HLH), a referral hospital in a rural area in Tanzania, has enabled advanced training for Haydom researchers, strengthening collaborative research capacity in clinical and academic contexts, and building diagnostic capacity in clinical microbiology.

Several collaborative studies over the last decades had documented a clear association between high levels of antimicrobial resistance (AMR) and a high burden of infant mortality in Sub-Saharan African countries [9]. The gut microbiome had also emerged as a new and highly relevant research area, and therapeutic modulations of the gut microbiome had shown promising results [10].

The research group at UiB has previously conducted investigations in Tanzania, primarily focusing on large urban centres, such as Dar es Salaam. These investigations included both observational studies involving paediatric populations [3, 4, 6, 8, 9, 11, 12] and a single interventional randomized controlled trial (RCT) targeting adults [13, 14]. The findings from these studies consistently demonstrated a high prevalence of severe infections associated with multidrug-resistant (MDR) bacteria. Consequently, the necessity of implementing preventive measures to prevent/reduce the incidence of sepsis/severe bacterial infections was recognized. Given the limited availability of data from rural regions and for the purpose of inclusivity, there was a compelling rationale for conducting this RCT in a rural setting, despite the anticipated increase in transportation costs related to recruitment of participants from their villages compared to urban environments. Of specific interest, several trials investigating the use of probiotics to improve infant health had been conducted over the last decade. The vast majority of these had been conducted in high-income countries (HICs) [15], and their main focus had been on reduction of necrotising enterocolitis, sepsis and mortality in preterm infants [16, 17]. All these results along with existing collaboration and partnership prompted the initiation of plans for a RCT in rural Tanzania on this topic, which aimed to evaluate the potential of probiotics in reducing mortality and morbidity in newborns, potentially through the mediation of decreased gut colonization by AMR bacteria.

### The collaboration and research team

In order to perform a large RCT with potentially impact to change guidelines, a multidisciplinary team with partners from both Tanzania and Norway with complementary competences was established. For this trial we specifically included researchers with competence and experience doing clinical trials in Africa and clinical researchers with a diverse experience from the clinical fields paediatrics/neonatology, microbiology and infectious diseases including AMR research. If possible, we had key persons in these clinical fields both from Tanzania and Norway. We also included scientific partners with advanced training in molecular microbiology and metagenomics.

It was clear that in addition to the necessary training and experience in performing clinical trials, local knowledge of culture and language was equally important. A long-standing collaboration between partners from UiB, University of Tromsø (UiT) and SUH in Norway and Tanzanian colleagues was key when setting up the team. Beyond technical expertise in clinical research, the importance of deep-rooted local knowledge, cultural sensitivity, and trusted community relationships was immediately evident. The experienced and trained local field workers were essential for success when aiming to perform a large RCT in a rural setting of a LMIC.

While the original plan envisioned a multisite approach including an urban and rural population in Dar es Salaam which is the largest and commercial city and Haydom which is in a rural region of Tanzania, respectively, a pivotal planning meeting —between investigators from Stavanger, Bergen and Haydom, shifted the course of the project. Haydom Global Health Research Centre had long experience with clinical trials performed in this region [18–20]. Drawing from their extensive experience, Haydom researchers convincingly argued that HLH, despite its rural setting, had the infrastructure, field experience, and community trust needed to conduct the entire study independently. Unlike the complexity and competing priorities in urban centers like Dar es Salaam, Haydom provided a more cohesive and controlled research environment, supported by years of successful community-based studies. This stability, coupled with low population mobility and a defined geographical area made it the ideal setting for a follow-up study of this scale and importance that could generate findings that are relevant for similar rural LMIC settings. In aftermath we also believe that the high (97%) retention rate at 6 months the ProRIDE trial would not have been possible if we had included a second urban study site in Dar es Salaam. Obviously with two sites we could have increased generalisability, but that would have come with a great threat of a much larger number of missing cases at follow-up which, increasing the risk of attrition bias.

Since 2019, at HLH there was already an ongoing research initiative, including capacity building for improved microbiology services. The director of HLH had recently finished a PhD in collaboration with SUH [21, 22]. HLH had an experienced pediatrician who knew the local clinical setting ensuring that the trial was accepted both in the hospital and in the surrounding catchment area. A medical doctor at Haydom Global Health Research Center, was employed to oversee the whole trial including contact with regulatory authorities and day to day follow-up of recruitment and study visits [23]. A field supervisor at Haydom Global Health research center supervised the field works and was essential in the day-to day work with the trial. The qualifications required for field workers in this study were a mandatory minimum of ordinary level secondary school education. Additionally, some candidates possessed diplomas in nursing, education, or clinical trial management. Upon recruitment at HLH, these individuals underwent two foundational training sessions: one focused on research ethics and the other on Good Clinical Practice (GCP). Prior to initiating participant recruitment, field workers received comprehensive training on the specific study protocol. This training ensured that they were well-versed in the standard operating procedures. An experienced microbiologist at MUHAS in Dar Es Salaam, who had finished a PhD in Bergen, Norway, was contributing to both microbiology and regulatory issues. Finally, an experienced Tanzanian researcher and microbiologist, having positions at UiB and at Liverpool School of Tropical Medicine, had a key role in the trial team with monitoring the entire ongoing study and frequently visiting the site for consultation.

### Haydom Lutheran Hospital (HLH)—the study site

**Fig. 1:**
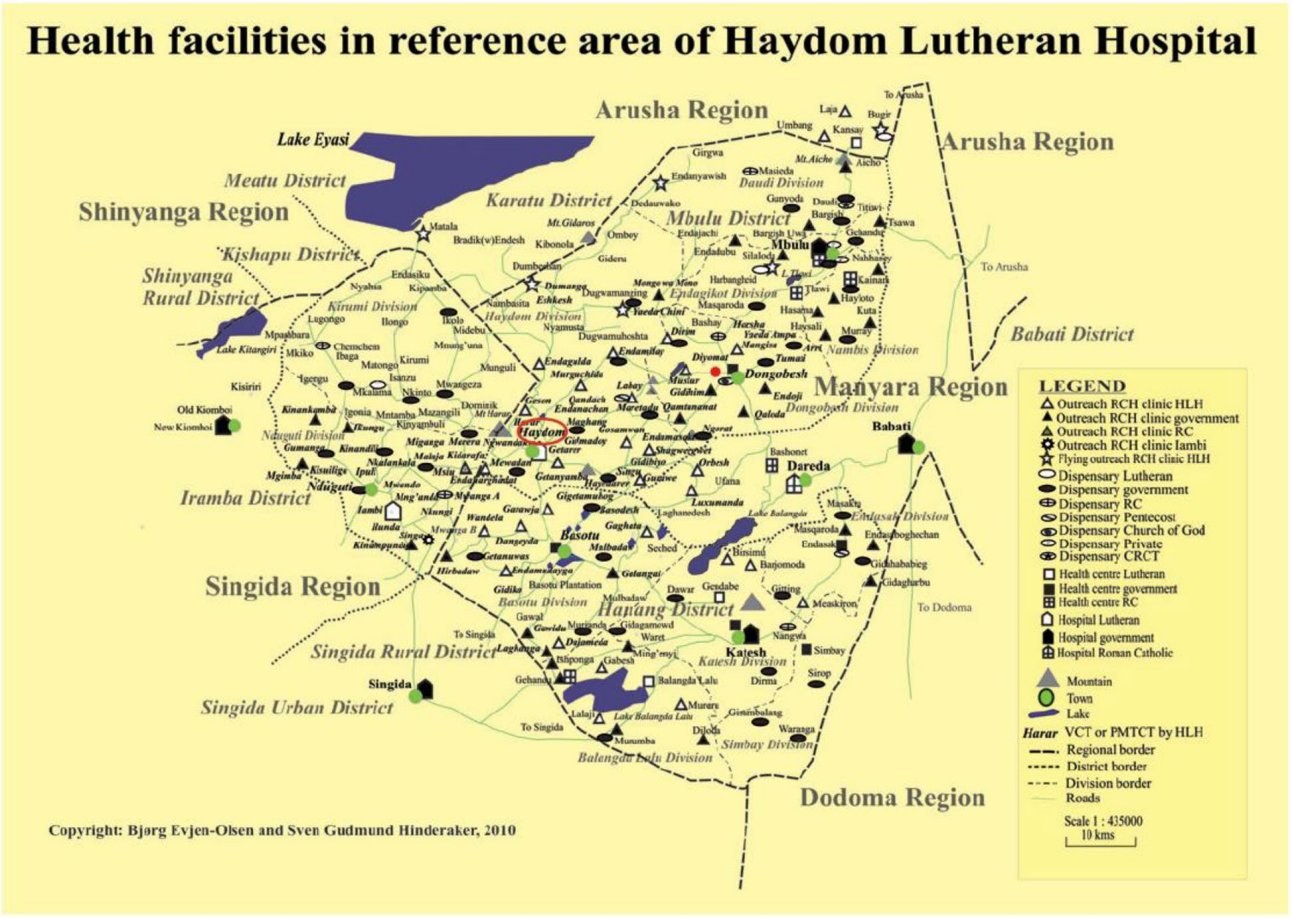
Map of villages in the study area surrounding Haydom Lutheran Hospital (HLH) circled in red, Tanzania. Adapted from Bjørg Evjen-Olsen and Sven Gudmund Hinderaker, 2010

HLH is a regional referral hospital located in Mbulu district, at the western end of the Manyara region in the North-Central Tanzania, about 300 km south-west from regional centre Arusha (Fig. 1). HLH functions as a level II regional referral facility and serve as a major referral centre for surrounding rural areas. It provides essential healthcare services to an estimated 900,000 people within its immediate catchment area and up to 5.7 million people across four regions and seven districts in its extended referral zone. Annually, there are around 3,900 deliveries in the hospital.

Despite its rural location, HLH has a strong and well-established research infrastructure. Over the past ten years the hospital has been involved in community research and large projects such as the MAL-ED study [19] and Helping Babies Breathe/Safer Births projects [24]. All these research programs underscore HLH's commitment to advancing neonatal and maternal healthcare through evidence-based research and intervention programs and at the same time build trust between the hospital and the surrounding communities.

### Community engagement

A stepwise community engagement process was undertaken prior to embarking on the ProRIDE trial. The process started with government and health administration approvals at regional, district, and ward levels, followed by meetings with all village leaders in the catchment area to ensure community acceptance and approval. Furthermore, a total of 12 meetings were organised during 18 months of trial recruitment and follow up. To assess the community’s attitude and acceptance of the intervention before the study began, we conducted focus group discussions with women caring for infants. The primary goal of the sensitization process was to raise awareness, address concerns, and strengthen community trust in the trial. We believe that the hospital’s well-established relationship with the local community, along with extensive engagement and educational efforts, was instrumental in successfully recruiting 2,000 infants within 11 months, and achieving a high retention rate of 97% at the 6-month follow-up in the ProRIDE trial.

### Ethical equipoise and regulatory approvals

There was a genuine uncertainty about the effect of the probiotic intervention on morbidity and mortality of the target population in this trial. Thus, it was an ethical equipoise, supporting the need for such a RCT. A study from India was conducted where more than 4500 term-born infants were randomized to receive either the combination of probiotic and prebiotic is coined “synbiotic” or placebo [25]. This simple intervention reduced the composite outcome of severe infections and/or death by 40%, from 9.0% in the placebo group to 5.4% in the synbiotic group. This trial was performed in a different continent with a different probiotic product combined with a prebiotic [25]. The remarkable benefits observed in India needed further evaluation in large-scale trials before the approach could be adopted as standard care for newborns. Especially since studies of this magnitude remain scarce in low- and middle-income countries (LMICs). Notably, there was no established preventive strategy against infant gut colonization with MDR bacteria (e.g. ESBL-E), a growing concern as gut colonization may proceed to invasive infections and sepsis among infants. Particularly in LMICs where AMR is widespread and treatment options of bacterial infections are limited, colonization and subsequent infections with ESBL-E and other MDR bacteria are a significant cause for neonatal mortality. Since the Indian trial did not specifically address this issue, further research was crucial to determine whether probiotics could serve as an effective preventive intervention to reduce gut colonization and infections with ESBL-E, and thus mortality. Conducting the ProRIDE trial did therefore not breach an ethical equipoise on this point. Infants included in the ProRIDE trial did not undergo any painful or invasive procedures, in particular no extra blood samples. Even though the study products are not considered to be medicinal drugs, and studies have so far not revealed any serious adverse effects**,** study participants were also insured, if unforeseen related side effects of the probiotics develop. Moreover, parents of the included infants– both in the intervention arm and control arm – were informed that if their infants would need medical care and hospitalization for any illnesses, they would receive standard care treatment at HLH, including study-funded optimal assessment of a potential infection with blood culture and C-reactive protein (CRP) analyses.

The ProRIDE trial was approved by local regulatory authorities, and both the National Institute of Medical Research in Tanzania (NIMR) and Tanzania Medicines and Medical Devices Authority (TMDA). It was a great advantage to have experienced Tanzanian researchers in our research team, who also knew the national regulations and how to respond to questions from the authorities. Without this local/national expertise in our research team, the trial would not have been possible to perform. Since the study was funded from Norway, we also sought and received approval from the Regional Committee for Medical and Health Research Ethics Western Norway, Norway.

### The research protocol – a pragmatic approach

We believe that a key factor in the successful completion of this trial was the deliberate effort to keep the intervention and study structure simple and practical. We chose to collect stool samples, a specimen which is non-invasive and easy to collect. We deliberately did not include any blood sampling of the study participants, except for blood culture and CRP if the child was admitted and infection was suspected. We certainly acknowledge that biological material, such as blood, is of high value for many studies. However, blood sampling may be difficult in infants, it inflicts pain on participants, and it increases the risk of poor recruitment. Blood sampling would also have vastly increased the cost of the trial by requiring more study nurses/field workers and expensive storage, transport and analyses. We believe that a more complex design would have hampered the study and potentially reduced generalizability. Moreover, the chosen multistrain probiotic product was one of very few commercially available products and was easy to administer as droplets [26].

Caregivers in our study administered the study drug at home, resembling real-life therapy and increasing generalisability. Many RCTs on intake of drug or nutritional products require that caregivers are assisted in administering the investigational product and that administrations are done under supervision. This was not feasible in our study with a four-week long period for administration. We therefore rather asked caregivers regarding adherence at follow-up visits, specifically how many days the investigational product had not been administered. We also monitored feacal samples for differences in gut microbiota composition, as an indirect proof of probiotic intake. Finally, at the end of the intervention, field workers checked the bottles and made a note if it was empty or not.

Another essential factor contributing to the successful completion of this clinical trial was the thorough planning phase conducted prior to the study's initiation. The research team from Norway undertook two planning visits and engaged in discussions with both the research team and the leadership of HLH during the year 2019. These meetings facilitated the assessment of the study site's appropriateness, including the evaluations of the research team at HLH as well as the clinical and laboratory capacities available. Through these discussions, the team identified specific areas requiring strengthening, particularly in laboratory infrastructure development and the provision of training in blood culture techniques and antimicrobial susceptibility testing. Furthermore, the effective planning of the trial, concerning the stool sampling process and the development of a comprehensive electronic Case Report Form (CRF) utilizing the REDCap platform was critical to success. The systematic organization of field workers, and the ongoing, supervision of the trial at the study site by our microbiologist were also essential components contributing to the overall efficacy of the trial.

### Budget and funding

Performing a large RCT has high costs, and funding limitations threaten both the initiation of studies and the timely completion according to protocol. In this clinical trial approximately 50% of the allocated budget was designated for expenditures related to the study site in Haydom. This budget was utilized for various purposes, including human resources, capacity building through the procurement of laboratory equipment, and costs associated with transport of field workers to various villages for data collection and follow-up with study participants. The remaining portion of the budget was utilized for shipment of samples, acquisition of laboratory consumables, investigational products, and laboratory analysis such as ESBL-E screening and microbiome assessments of fecal samples, among others.

### Challenges faced before the trial started and how we overcame them

The ProRIDE trial was initially planned to start in 2020. However, study start was postponed due to several reasons. The onset of the COVID-19 pandemic and subsequent lockdown measures was a main reason for postponement of study start until February 2022 [27]. During this period, the study faced several critical challenges, including the financial burden of compensating field workers and researchers despite the temporary halt in study activities. Additionally, procured stool collection kits and investigational products, including probiotics and placebo, expired due to the delay and led to increased costs as new products had to be procured. These setbacks contributed to a significant rise in overall trial expenses. Despite these challenges, the trial successfully commenced in February 2022.

The ProRIDE trial included an investigational product (probiotic) and placebo. However, the probiotic investigational product is classified as a food supplement rather than a pharmaceutical drug in Europe. The product is produced in South Africa, under strict quality regulation, on behalf of the manufacture in the United Kingdom [1, 2]. Due to its classification as a food supplement, a Certificate of Good Manufacturing Practice (GMP) is not typically issued. Nonetheless, since the study is registered as a clinical trial, regulatory authorities in Tanzania mandated the submission of a GMP certificate prior to granting approval for the trial, which was submitted. The necessity of clarifying the distinctions between the manufacturer’s classification of the product and the regulatory requirements resulted in extended delays in obtaining the requisite approval to initiate the trial.

The process of training and recruiting field workers required considerable time to ensure they were well-equipped with the necessary skills for participant enrolment, data collection, and community engagement. Additionally, logistical arrangements, such as acquiring and maintaining motorbikes for field mobility, organizing transport for study personnel, and ensuring adequate supplies for remote site visits, added to the complexity and duration of the preparatory phase. However, despite the delays caused by the pandemic, this unexpected setback provided an opportunity to conduct more comprehensive training of field workers, ultimately strengthening the team’s capacity and preparedness. In this sense, the pandemic became a blessing in disguise, allowing for a more thorough and well-structured trial setup.

## The recruitment process

The study recruited newborns born in HLH and newborns born at small health facilities or at home in the surrounding area. Pregnant women in their third trimester were approached either at their residences or during their prenatal visit at HLH. These participants received both oral and written information regarding the study and were solicited for their consent to allow their future child to partake in the research. A research assistant, fluent in the participants' native language, provided a comprehensive overview of the study's objectives, emphasizing the significance of the research and detailing the procedures that would be undertaken throughout the study period. Participants were thoroughly informed about the administration of the investigational product or placebo, which involved taking five drops orally once daily until the bottle was empty, spanning a duration of four weeks. The necessity of adhering to the complete dosage regimen of the investigational product or placebo throughout the four-week intervention period was also clearly articulated. Both verbal and written informed consent forms were utilized to ensure understanding and agreement. Should the parent/caretaker consent to the child's involvement in the ProRIDE Trial following this briefing, they were asked to provide written consent for the screening and enrolment of the newborn in the study. For caretakers who lacked literacy skills were provided with the option of utilizing an "impartial witness." This individual, selected by the caretaker, possesses the ability to read and write and serves as a representative during the consent process. When the field worker presents the consent form to the caretaker, the impartial witness is present to listen and concurrently read the document. Upon completion of the reading, the witness verifies to the caretaker that the content conveyed aligns precisely with the text of the form. Subsequently, the witness affixes their signature as a confirmation of this verification. After delivery and upon inclusion in the trial, the parent/caretaker received a specially designed information card pertinent to the ProRIDE Trial and a copy of the signed consent form.

For mothers who gave birth at the hospital, attending medical personnel—including doctors and midwives—identified those who had previously consented to participate in the study and relayed this information to the research assistant, who managed study logistics. Additionally, research nurses were stationed in the maternity ward to actively monitor deliveries and ensure timely enrolment of participants. In the community, health workers associated with the study played a crucial role by notifying the study team when a mother who had consented to participate was referred to the hospital for delivery or had given birth in a small health facility or at home, ensuring comprehensive follow-up and inclusion in the study. After delivery, the newborn was screened for eligibility to participate in the study. If the infant met the inclusion criteria, enrolment ensued, and the newborn was randomly assigned to receive either the active probiotics or the placebo. This intervention commenced on day 0–1 for infants delivered in the hospital or on day 0–3 for those born in small health facilities or at home. The mother was once again provided with a detailed explanation and demonstration regarding the administration of the study product.

### Strategies to increase recruitment and retention after the trial had started

**Fig. 2:**
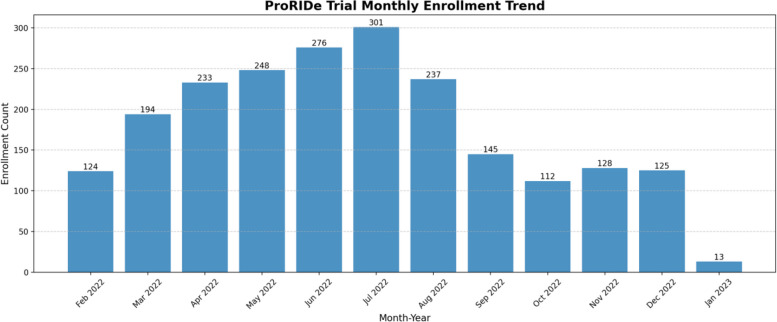
Monthly recruitment of participants

Despite the delayed start of the trial and budget constraints, we remained committed to our target sample size of 2,000 infants. To accelerate recruitment, the study team strategically expanded our team by increasing the number of field workers and community health workers, ensuring a more efficient outreach and recruitment process (Fig. 2).

Originally planned as a 12-month recruitment period, these efforts allowed completion of enrolment one month ahead of schedule, demonstrating the effectiveness of the adaptive approach and strong community engagement.

**Fig. 3:**
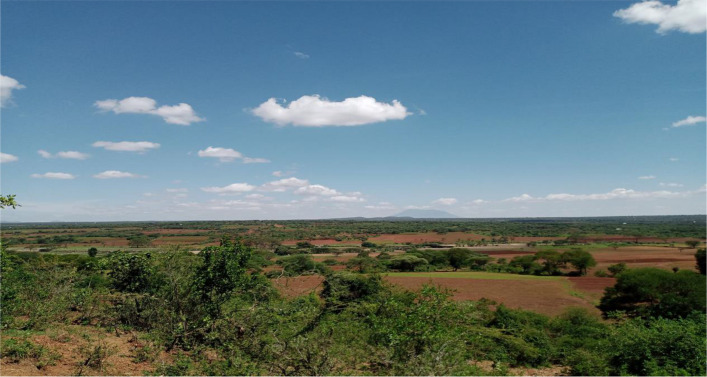
Remote villages of Haydom

Based on culture and tradition of the Haydom society, the pregnant women could not give consent of their future babies to participate in the study without the consent of their husbands/partners. This rural area is largely depending on farming activities; therefore, men go to their farming activities very early in the morning. Due to the need of men to also give consent for the future babies to participate in the study, field workers needed to start working at 5am (Fig. 3) to reach far villages to obtain consent before men go for work/farming.

To expedite the recruitment process, the identification of pregnant women was conducted through a systematic approach. Community health workers were tasked with notifying field workers about the presence of pregnant women within their respective areas. Subsequently, field workers engaged in direct communication with these pregnant women to provide information regarding the trial and to inquire about their willingness to participate in the study.

The substantial presence of 30 field workers and 36 community health workers facilitated not only the acceleration of participant recruitment but also ensured the timely execution of follow-up visits for sample collection and the reporting of adverse events. This organizational structure was instrumental in guaranteeing that samples were collected within the designated timeframe and that adverse events were reported promptly.

Due to the size of catchment area, which extended 30 kms around HLH, and the considerable distance between villages and residences, the expansion of field workers and community health workers necessitated an increase in the transport budget to access remote villages. This expansion required the utilization of additional vehicles and motorcycles to facilitate transport of field workers engaged in recruitment, follow-up visits, and sample collection. Consequently, this increase in transport resources contributed to a rise in the overall costs associated with the trial. Furthermore, we increased the number of laboratory personnel from two to five to facilitate the processing of an increased volume of samples within a compressed timeframe.

### Data capture during the study

Data was managed by using the REDCap® (Research Electronic Data Capture) tool hosted by UiB, with information captured and recorded in electronic case report forms (eCRFs) [28]. For enrolled infants, four different eCRFs were used to collect clinical and demographic information – three for the study visits and one for adverse events (AE) and unscheduled visits due to hospitalization or outpatient clinic attendance. Tablets with REDCap® software were provided to field workers and investigators. Passwords were created for each research assistant and investigator to get access to the study file in REDCap. Research assistants had only access to fill in the respective CRFs of the study participants they recruit at each visit, and no access to change any information entered in REDCap. All data with identifiers were stored on firewall-protected secure servers, separately from clinical and microbiological data. All staff members involved in data collection received training in utilizing the REDCap tool, as well as training in Good Clinical Practice (GCP) guidelines. Quality assessment of the data was done by the internal clinical staff and consent forms and electronic CRFs were checked by external monitors.

### Capacity building at HLH and shipment of samples from Tanzania to Norway

The research group undertook a capacity-building initiative at the local laboratory by enhancing its infrastructure with the acquisition of essential equipment, including a −80 °C freezer, a small refrigerator/freezer, equipment for preparing agar/media, a CO_2_ incubator, and a blood culture incubator. Training for the laboratory personnel was conducted by microbiologists from Norway, focusing on critical competencies needed for blood culture diagnostics such as media production, sample processing, including bacterial identification, antimicrobial susceptibility testing and quality control. Nonetheless, ESBL-E screening and more advanced analysis, such as microbiome sequencing, whole genome sequencing, metabolomics etc. could not be performed locally due to lack of capacity, equipment’s and expertise, necessitating the shipment of samples to Norway for processing.

The logistical aspects of this undertaking were significantly complicated by difficulties in the procurement of dry ice, in conjunction with the elevated expenses associated with the transportation of samples utilizing dry ice. The research team from UiT visited the trial site to organise the first shipment of samples in September 2022. Due to the substantial volume of samples collected, the research group organized three separate shipments. Despite the logistical difficulties encountered, proper planning and preparation of sample shipment before the time of shipment enables the team to successfully ship samples which arrived in Norway in good condition.

As part of capacity building, the research group successfully obtained funding for a PhD position at UiT-The Arctic University of North Norway in Tromsø. The ProRIDE local study coordinator, Museveni Justine, embarked on his PhD studies in 2024 aiming to enhance future research capacity both for himself and for Haydom, where he lives with his family. He is currently (autumn 2025) analysing stool samples, including extraction of nucleic acids to evaluate stool pathogens and performing advanced enzyme-linked immunosorbent assays (ELISAs) to assess gut inflammatory markers.

### Dissemination of results to caretakers

Clinical trials are essential for medical advancement, benefiting both low- and high-income populations worldwide. However, there is a significant disparity in how these benefits are distributed, especially when it comes to sharing the results of studies with those who participated in them. Marginalized and underserved communities often take part in these trials, contributing invaluable data that drives scientific progress. Yet, these communities frequently remain unaware of the outcomes of the studies they were involved in, primarily due to a lack of effective communication from researchers [29–31]. This limited access to information prevents participants from understanding the broader implications of their contributions, including potential health benefits or risks identified in the research.

Dissemination of clinical trial results to participating communities remains a well-documented challenge, particularly in resource-limited settings where feedback mechanisms are often underdeveloped and logistical constraints are common [32, 33]. Recognizing this, the ProRIDe trial team prioritized community-level communication as an integral part of the research process. In August 2025, we successfully conducted a centralized dissemination meeting at HLH, involving community leaders and representatives from all participating villages and faith-based leaders. This achievement was made possible through the strong collaboration between HLH, local authorities, and academic partners from Norway and Tanzania. The same community engagement structures that had supported recruitment and sensitization were reactivated to deliver the findings, ensuring continuity and trust. The meeting, conducted in Swahili and supported by user friendly illustrations, translated complex results on infant morbidity, mortality, and reduction of AMR bacteria carriage into clear, accessible messages. The exercise was well received, with community representatives expressing appreciation for being informed of the outcomes and for the hospital’s ethical commitment to transparency. This experience demonstrates that, although dissemination in rural and resource-limited contexts can be challenging, it is both achievable and impactful when grounded in collaboration and community trust.

## Summary and conclusions

In this randomized clinical trial, we successfully completed the recruitment of 2,000 participants within a span of 11 months, despite encountering various challenges both prior to and during the trial. Additionally, the trial exhibited a high retention rate of 97%, with stool samples collected from 95% of the study participants.

Several key elements were important for the success of this trial. A multidisciplinary team with broad expertise, and with partners from both high- and low-income settings was essential. Thorough preparations and training of local field works was also extremely important. We believe that our “pragmatic study design” including a restricted number of samplings, easy to use electronic case report forms and “real-world” home administration of the investigational products were important. Finally, the trial would not have been a success without robust collaboration and teamwork, and engagement and trust of the communities, throughout the process.

## Data Availability

Not applicable.

## Abbreviations

AE/SAE: Adverse events/serious adverse events
AMR: Antimicrobial resistance
BSI: Bloodstream infection
CRF: Case report form
ESBL: E-Extended-spectrum beta-lactamase Enterobacteriaceae
eCRF: Electronic case report forms
GCP: Good Clinical Practice
HLH: Haydom Lutheran Hospital
LMICs: Lowand middle-income countries
MUHAS: Muhimbili University of health and Allied Sciences
NIMR: National Institute of Medical Research in Tanzania
RCT: Randomized controlled trial
REK: Regional Committee for Medical and Health Research Ethics of Western Norway
UiT: University of Tromsø
UiB: Universoty of Bergen
WHO: World Health Organization
